# Additional Insights Into Problem Definition and Positioning From Social Science: Comment on "Four Challenges That Global Health Networks Face"

**DOI:** 10.15171/ijhpm.2017.108

**Published:** 2017-09-10

**Authors:** Kathryn Quissell

**Affiliations:** Department of Health Sciences, Sargent College, Boston University, Boston, MA, USA.

**Keywords:** Global Health Networks, Advocacy, Problem Definition, Framing, Effectiveness

## Abstract

Commenting on a recent editorial in this journal which presented four challenges global health networks will have to tackle to be effective, this essay discusses why this type of analysis is important for global health scholars and practitioners, and why it is worth understanding and critically engaging with the complexities behind these challenges. Focusing on the topics of problem definition and positioning, I outline additional insights from social science theory to demonstrate how networks and network researchers can evaluate these processes, and how these processes contribute to better organizing, advocacy, and public health outcomes. This essay also raises multiple questions regarding these processes for future research.

Much effort is put into rigorously monitoring and evaluating the effectiveness and impact of global public health interventions. We should be putting a similar level of effort into studying and ensuring the effectiveness of global health advocacy and governance. These processes can also have an impact on health outcomes by determining what issues make it onto political agendas, but they receive far less attention. Advocacy, policy, and governance processes are complex, but one factor important to all of them is how problems are understood and framed both internally among coalition members and externally to policy target audiences.^1^ However, attention to problem definition and positioning is still limited in much of the scholarship on public health policy for many health issues.^2^ Social science theories and methods can help address this shortcoming.

As outlined in Jeremy Shiffman’s recent editorial^3^ and Jale Tosun’s commentary,^4^ the proliferation of global health networks and the subsequent complexity of the global health governance landscape are examples of some of the current challenges that can be better understood, and hopefully managed, through the use of social science theories and research methods. Shiffman’s article draws together the findings of eight different qualitative case studies of global health networks using ideas from international relations, sociology, public administration, and other fields to answer important questions around what makes some global health networks more effective than others, and it presents a concise set of four challenges that networks will have to tackle in order to maximize their opportunity for impact. These challenges include coalition-building, network governance, achieving consensus on problem definition, and creating an inspiring public positioning of their issue of concern. All four challenges present various complexities individuals working in these networks may encounter, but I would like to focus here on problem definition and positioning as they are closely related, and as they are central to effective coalition-building, governance, and advocacy. To do so I present several additional social science theories and concepts that have a lot to offer as a means of assessing these processes in prospective network organizing, with the goal of improving advocacy and public health outcomes.

First of all, as Shiffman describes, problem definition relates to how members of a network come to understand an issue, and he groups both the problem and its solutions under the same challenge. However, other social science scholarship describes problem and solution definition as distinct streams.^5^ These processes may be linked, but they can also be separate, making it useful to think of them as two different steps in the policy process. For example, the global HIV/AIDS community is arguably one of the most effective global health networks. The amount of attention and resources raised far surpasses that of other global health issues.^6^ This network is also made up of multiple sub-networks representing diverse constituencies and viewpoints. There are the AIDS Coalition to Unleash Power (ACT UP) and Treatment Action Campaign (TAC) civil society and social justice organizations who fight for the rights of people living with HIV/AIDS, and there are also faith-based organizations who work to prevent suffering and early death. There may be some overlap in problem definition between these organizations, but it is hard to believe that gay rights advocates and evangelical Christian groups see the problem of AIDS in exactly the same way. Many solutions for addressing AIDS are linked to these various problem definition viewpoints, and have led to significant debate and conflict, such as whether or not prevention programs should emphasize condom distribution and comprehensive sex education or abstinence only. One of the reasons this broad coalition of diverse actors was able to effect change was the coalescence around antiretroviral treatment (ART),^7^ a solution that individuals and organizations with many different problem definitions could all agree to supporting and implementing. This solution avoided many of the conflicts surrounding appropriate sexual behavior that could have exacerbated network fragmentation and conflict. Particularly since broader coalitions have also been found to make more effective networks,^3^ there is likely a need for networks to figure out strategies for increasing both diversity and collaboration. Agreement on problem definition may not be necessary to this process, but this is an empirical question deserving further study.

In terms of practice, better communication between network members and greater time spent analyzing areas of disagreement could be of significant benefit to networks experiencing conflict. For example, several questions networks should ask of their members are: what exactly are the disagreements we are having? Is it problem definition, solution definition, both? Could we overcome conflict surrounding problem definition with better agreement around solution definition? Are there places we could compromise, or are there core beliefs different members hold that cannot easily be changed?

These questions also relate to positioning the issue for audiences outside of the network. When targeting donors and health and other ministries, networks typically rely on the problem and solution definitions they create internally, and not necessarily on the way their targets see the issue. Research in social psychology and social movement scholarship has found that alignment in framing is important for persuasion and the adoption of new policies.^8-11^ The more thinking and strategizing networks have done internally on how to broaden the coalition of supporters, the more prepared they will be for external audiences. This process is referred to as strategic framing, which advocates can use to emphasize different values or norms surrounding the problem and solution with the goal of matching the values or norms held by policy gatekeepers.

However, strategic framing and effectively positioning some issues may be more difficult than others, and this can depend both on characteristics of the issue and characteristics of the advocates. For example, a substantial body of research on US policy has found that the population targeted by policies has an effect on the likelihood governments will pay attention to the problem and whether or not adequate resources will be allocated,^12^ finding that policy target groups with limited political power, and particularly those who are seen negatively, are more likely to see constrained, or non-existent, policy benefits. Advocates can have very little influence on this particular issue characteristic unless they target their advocacy at strengthening the political power of groups and/or changing the social valuation of these populations from negative to positive (such as overcoming group stigma). This is a significantly more complex process of social transformation and not a simple marketing exercise, but it might be necessary for advocacy success on certain issues. Within a diverse network there is also likely to be a range of perspectives on short-term vs. long-term goals, such as these, that will influence problem and solution definition, as well as public positioning. Clarifying these positions within networks can therefore help with better strategic planning.

Lastly, it is also important for global health networks to consider the consequences of how problems and solutions are framed. To use the example of HIV/AIDS again, while mobilizing around universal access to ART is one explanation for advocacy success, other studies have found that framing the issue externally as a problem of vulnerable women and children has helped to increase donor aid and to direct more policies and resources at ART for women.^13^ This external positioning is consequential because it demonstrates how framing women as vulnerable helped to increase resource provision. Vulnerability implies innocence and a lack of responsibility for the stigmatized behaviors contributing to transmission,^14^ which mitigates HIV/AIDS related stigma and improves the social valuation of women. However, it also creates another policy target population responsible for spreading the disease – men. While the vulnerability frame may accurately reflect the circumstances of many women worldwide, its dominance among global programs may have had the unintended consequence of neglecting men who are also in need of treatment.^15^ Ultimately what this frame does is deflect the stigma of HIV/AIDS from one group to another. It may have contributed to policy attention and resources in the short-term, but in the long-term it does not address the underlying problem of stigmatization, and it may be putting lives at risk. Currently, women are about 51% of the HIV+ population globally, but 58% of AIDS-related adult deaths are among men.^16^ If men are framed as at-fault and as disease-vectors, what impact does this have on how healthcare providers engage with them? Is this narrative having a negative influence on men’s health seeking behaviors? Is it contributing to worse health outcomes? These are all questions worth investigating in more detail.

Problem and solution definition and the external positioning of issues are key processes in effective advocacy, and how these processes are carried out can have an important influence on coalition building, network governance, persuasion, strategic planning, and the short-term and long-term impacts of public health programs. Scholars and practitioners should analyze and critique these processes more systematically, and social science theories and research methods can help. The research synthesized in Shiffman’s editorial are all examples, but there is a lot more that we need to learn about these complex processes.

## Ethical issues

Not applicable.

## Competing interests

Author declares that she has no competing interests.

## Author’s contribution

KQ is the single author of the paper.
